# Defining hip cartilage repair: a modified delphi study to establish the Magnetic Resonance Evaluation of the Repair of Cartilage in the Hip (MERCH) score

**DOI:** 10.1186/s40634-023-00676-y

**Published:** 2023-12-05

**Authors:** The Magnetic Resonance Evaluation of the Repair of Cartilage in the Hip (MERCH) Investigators and The Canadian Hip Preservation Research Collaborative (CHIPR), Dan Cohen, Pierre-Olivier Jean, Kelly Johnston, Nicole Simunovic, Pouyan Ahangar, Paul E. Beaulé, Etienne L. Belzile, Jordan Buchko, Sasha Carsen, Jas Chahal, Hema Choudur, Ryan Degen, George Grammatopoulos, Waleed Kishta, Devin Lemmex, Parth Lodhia, Marie-Lyne Nault, Naveen Parasu, Thierry Pauyo, Kevin Smit, Daniel Whelan, Geoffrey Wilkin, Kevin Willits, Ivan Wong, Olufemi R. Ayeni

**Affiliations:** 1 Department of Surgery, Division of Orthopaedic Surgery, McMaster University, Hamilton, ON Canada; 2 McMaster University Medical Centre, Hamilton, 1200 Main St West, 4E15, ON L8N 3Z5 Canada

**Keywords:** Hip, Cartilage, Repair, Reconstruction, MRI

## Abstract

**Purpose:**

To develop a standardized scoring system to evaluate pre- to post-operative repair or reconstruction of hip cartilage using magnetic resonance imaging (MRI).

**Methods:**

A two-phase modified Delphi study was conducted. Phase 1 involved a survey with suggested criteria and diagrams to define various stages of articular cartilage repair and phase 2 involved an expert consensus meeting that discussed the survey responses and voted on final scoring criteria. The survey was emailed to members of the Canadian Hip Preservation Research Collaborative (CHIPR) and respondents included both board certified orthopedic surgeons as well as musculoskeletal radiologists.

**Results:**

Overall, there were 17 survey respondents from Canada and most (47%, 8/17) participants agreed that the *minimum* MRI protocol needed to evaluate cartilage repair was a 3.0 T MRI and 94% (17/18) agreed that the minimum time post-operatively that they felt they would be able to accurately evaluate cartilage repair on an MRI was 12 months. Following phases 1 and 2, the final Magnetic Resonance Evaluation of the Repair of Cartilage in the Hip (MERCH) score was developed with 7 domains, 3 criteria per domain: 1) volume fill of cartilage defect, 2) integration into adjacent cartilage, 3) surface of the repair tissue, 4) structure of the repair tissue, 5) bony overgrowth, 6) subchondral changes, and 7) delamination. The score ranges from 60 (optimal) to -20 points (worst/none).

**Conclusions:**

This consensus project established a new MRI scoring system to evaluate post-operative cartilage restoration of the hip. The implementation of the MERCH score is essential in our ability to guide patient management and expectations in a rapidly evolving field and will help with standardizing our evaluation of cartilage repair in future research trials.

**Level of Evidence:**

Level II Diagnostic.

**Supplementary Information:**

The online version contains supplementary material available at 10.1186/s40634-023-00676-y.

## Background

Articular cartilage defects of the hip typically do not heal due to the avascularity of cartilage and low proliferative capacity of articular chondrocytes – the producers of the functional extracellular matrix of articular cartilage [2, 14]. Moreover, focal articular cartilage defects, if left without repair, can develop into larger generalized lesions that cause significant pain and dysfunction. Perhaps more importantly, these generalized lesions also have the potential to hasten the early development of osteoarthritis (OA), which is irreversible [4]. While hip replacements are generally safe and effective [11], young adults that have hip replacements are at risk of poorer outcomes and early failure [1]. Currently, there are many options for the repair and reconstruction of cartilage, including chondroplasty, delamination repair, microfracture, autograft and mosaicplasty, osteochondral allografts, and autologous chondrocyte implantation [7, 9, 20].

Given the variation in chondral damage, as well as the repair techniques, broad classification of cartilage status is not very descriptive for identifying patient outcomes and reoperation strategies. The International Cartilage Repair Society (ICRS) classification was developed to classify cartilage status, but is limited to just four categories, ranging from normal to severely abnormal [10]. There are two main scores used for the classification of cartilage status specific to the hip: the Scoring Hip Osteoarthritis with Magnetic Resonance Imaging (MRI) (SHOMRI) and the Hip Osteoarthritis MRI Scoring System (HOAMS) [12, 13, 16, 19]. However, these scoring methods do not evaluate the repair and integration of hip cartilage. The SHOMRI and HOAMS comment on the thickness of the cartilage remaining, but do not provide details on the type of repair and integration that has occurred from pre- to post-operatively. The Magnetic Resonance Observation of Cartilage Repair Tissue (MOCART) Score was developed to evaluate cartilage repair, but in the knee [18]. This study has developed the novel Magnetic Resonance Evaluation of the Repair of Cartilage in the Hip (MERCH) Score whose goal is to help with describing patient outcomes and the effectiveness of cartilage repair strategies in hip arthroscopy.

As the only score known to evaluate cartilage repair in a joint using MRI, we aimed to adapt the MOCART score to develop hip-specific criteria to evaluate pre- to post-operative repair and/or reconstruction of hip cartilage with a two-phase modified Delphi study. Given the similar diagnostic accuracy and extensive clinical training amongst the hip arthroscopy community, we hypothesized that there would be a general consensus on MRI criteria defining post-operative repair and/or reconstruction of hip cartilage.

## Methods

**Fig. 1:**
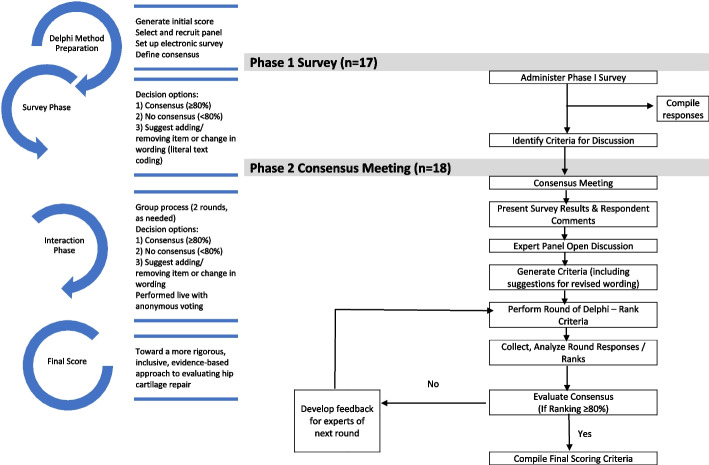
A flow diagram of the modified Delphi process for the MERCH study (Adapted from Oxford Consensus) [5]

The Hamilton Integrated Research Ethics Board (HiREB) approved the study protocol prior to initiation and all consensus participants provided written informed consent prior to the meeting (#15,021). A two-phase modified Delphi study was conducted involving a Likert/multiple-choice-based survey using diagrams to define various aspects of articular cartilage repair (Phase 1) and an expert consensus meeting that discussed the survey responses and voted on the scoring criteria (Phase 2) (Fig. 1). Throughout both phases of the study process, participants were asked to consider their responses in the context of global application, meaning the *minimum* MRI protocols and score criteria that could be used to evaluate cartilage repair given the disparities in access to diagnostic technology in low-, middle-, and high-income countries.

### Participants

The participants included members of the Canadian Hip Preservation Research Collaborative (CHIPR) who are board certified orthopedic surgeons with expert knowledge in the research and clinical assessment of patients with articular cartilage defects of the hip. Most CHIPR surgeons have a practice that consists of at least 50 hip arthroscopy cases per year in the last 5 years, had fellowship training in hip preservation, and are early to mid-career investigators. The Principal Investigator also identified 2 Canadian musculoskeletal radiologists with similar experience in radiological assessment of the hip and research. The radiologist participants had to have > 80% practice focus and training in musculoskeletal imaging. Participants were invited to participate in both the survey and consensus phases of the study via e-mail. Of these, 17 completed the Phase 1 survey and 18 participated in the Phase 2 consensus meeting.

### Phase 1: Survey assessment

Phase 1 of the modified Delphi process utilized a web-based survey that asked about demographic information of the respondents and the 7 proposed sections and criteria therein of the MERCH score. Participants were asked to rank how strongly they agreed with a statement or criteria using a 7-point Likert scale (Appendix 1). Open-ended questions were asked at the end of each section to determine if there were other important considerations for the scoring criteria. Research personnel compiled the responses and used literal text coding to look for commonalities in the open-ended responses and presented a summary to the consensus meeting participants in Phase 2 (Appendix 2). Descriptive measures of central tendency (means, proportions) and level of dispersion (standard deviations, ranges) were calculated for all criteria and presented to the consensus meeting participants.

### Phase 2: Consensus meeting

The MERCH scoring criteria was revised based on recommendations provided by the survey respondents and presented at the consensus meeting on January 14, 2023. In some cases, there were conflicting comments in the survey on how to handle specific criteria and consequently, all applicable comments were brought forward to the consensus meeting for discussion by the expert panel. The participants were also given the opportunity to consider revised wording of individual criteria. Following some discussion after reviewing each proposed section of the MERCH score, participants were asked to vote anonymously (using the poll feature of the Zoom virtual platform (Zoom Video Communications Inc., San Jose, CA, USA)) on each carefully worded criterion using a 7-point Likert scale (Appendix 1). Discussions were facilitated by a research staff member not directly involved in deciding the scoring criteria and continued until 80% of respondents agreed on inclusion or exclusion of a given criterion. If the 80% threshold could not be reached after a third round of discussion and voting, the result was to be marked as “no consensus”.

### Statistics

All calculations were reported using descriptive statistics, including means, proportions, and standard deviations (performed using SPSS version 28.0.1.0).

## Results

### Phase 1: Survey assessment

Overall, there were 17 survey respondents who performed a mean of 75 (SD 50.7) hip arthroscopy procedures per year (Table 1). Most respondents had a minimum of 4 years in practice (15/17, 88%), worked in an academic center (14/17, 82%) and were fellowship trained (16/17, 94%). Most participants were orthopedic surgeons (11/17, 65%), with the remaining being pediatric orthopedic surgeons (4/17, 24%) and radiologists (2/17, 12%), all with experience diagnosing and treating cartilage defects in the hip.

**Table 1:** Demographics of respondents

***N* = 17**	**No**	
**Annual hip arthroscopy procedures**		
Mean (SD)	75 (50.7)	
Median	65	
Maximum	200	
Minimum	10	
	**No**	**(%)**
**Perform hip arthroscopy procedures?**		
Yes	14	82.4
No	3	17.6
**Age**		
Less than 30 years old	1	5.9
31–40 years old	7	41.2
41–50 years old	7	41.2
51–60 years old	2	11.8
**Current position**		
Orthopaedic surgeon	11	64.7
Paediatric orthopaedic surgeon	4	23.5
Radiologist	2	11.8
**Country of practice**		
Canada	17	100
**Years in Practice**		
1–3 years	2	11.8
4–6 years	5	29.4
7–9 years	4	23.5
10–14 years	3	17.6
15–20 years	2	11.8
20 + years	1	5.9
**Clinical Setting**		
Academic Centre Hospital	14	82.3
Academic Centre Clinic	3	17.6
Community Hospital	2	11.8
**Fellowship or Additional Training**		
Yes	16	94.1
No	1	5.9

### Optimal MRI Protocol

Results from the survey demonstrated variability in what surgeons considered an appropriate MRI protocol to evaluate hip cartilage. When asked what they consider to be the *minimum* MRI protocol needed to evaluate cartilage repair, 47% (8/17) of respondents indicated a 3.0 T MRI and 41% (7/17) indicated a 1.5 T MRI with arthrogram. Some respondents agreed that the coronal plane alone was sufficient to evaluate articular cartilage repair, but most respondents felt that an additional plane should be incorporated into the score (10/17, 59%). However, the precise multi-planar sequence was variable with some suggesting a combined coronal/axial oblique (3/10, 30%), while others did not indicate an optimal plane (5/10, 50%). In terms of time to cartilage integration, most respondents agreed that the earliest timeframe post-operatively to accurately view cartilage repair on MRI was 6–12 months (13/17, 77%). However, there was disagreement regarding the optimal timepoint post-operatively to obtain an MRI with 47% (8/17) of respondents recommending 6–12 months post-operatively and 47% (8/17) recommending 13–18-months.

### MERCH Criteria

At the survey phase, the MERCH score was presented with 7 separate domains and 2 to 5 proposed criteria therein, similar to the MOCART score but with criteria relevant to the hip joint: 1) volume fill of cartilage defect, 2) integration into adjacent cartilage, 3) surface of the repair tissue, 4) structure of the repair tissue, 5) signal intensity of the repair tissue, 6) bony defect or bony overgrowth, and 7) subchondral changes (Appendix 2).

Overall, respondents indicated that there was a need to include multiple imaging planes, diagrams that were more “zoomed in” to better see the structural abnormalities being depicted, and more simplified criteria, which were incorporated into the score for evaluation in Phase 2. Refer to Table 2 for further information regarding the initial stage comments and modifications.

**Table 2:** Initial stage comments and modifications

Domain	Response/comments	Modifications
Volume Fill of Cartilage Defect	Lack of agreement on the definition of overfilling and the imaging appearance of complete cartilage delamination	Overfilling revised to represent anything over the defect borders and “complete delamination” revised to “complete void”
Integration Into Adjacent Cartilage	Split-like defects should be based on less than or equal to 2 mm	Score modified to define a split-like defect as one that is less than 2 mm
Surface of Repair Tissue	Some concerns regarding the imaging quality and ability to accurately depict surface irregularity using MRI	Size and details of images increased to more clearly demonstrate surface that is intact versus not
Structure of the Repair Tissue	Suggestion to adjust the depiction of heterogeneous repair tissue using closely dotted lines rather than a line	Heterogeneous image adjusted to utilize a closely dotted line for improved visualization
Signal Intensity of Repair Tissue	Would be difficult to evaluate consistently given disparities in MRI technology worldwide	Plan to discuss removal of this section entirely at consensus meeting
Bony Defect	Separate bony defect and bony overgrowth into separate categories and adjust cut off for definition of bony defect	Score adjusted to contain either “no bony defect”, “bony defect less than 2 mm” or “bony defect greater than 2 mm”
Subchondral Changes	Osteonecrosis should be its own category; subchondral cyst should be defined as present or absent and cut off for bone marrow edema should be 25% as opposed to 50%	Score adjusted to include category for osteonecrosis, subchondral cyst defined as present or absent and bone marrow edema cut off changed to 25%

### Phase 2: Consensus meeting

The original survey presented a more detailed and nuanced scoring system for respondents to evaluate (Appendix 1). Based on comments from the open-ended survey questions and discussion during the Phase 2 meeting, there was a general consensus that the score should be simplified to improve the ease of administration and the accuracy and consistency of the final score. Refer to Table 3 for further information regarding modifications at the consensus meeting.

**Table 3:** Consensus meeting modifications

Domain	Modification
Volume filling of cartilage defect	Now includes 3 categories representing “complete cartilage volume filling”, “any overfilling or underfilling”, and “complete void”
Cartilage Integration	Integration described as “complete”, “incomplete”, or “no integration”
Surface of Repair tissue	Surface described as “intact”, “not intact”, or “no repair tissue present”
Structure of the Repair Tissue	Structure defined as “homogeneous”. “heterogeneous”, or “no repair tissue present”
Bony Changes	Modified to focus on bony overgrowth as opposed to defects. New criteria included “no intralesional osseous overgrowth”, “some intralesional osseous overgrowth”, or “complete intralesional osseous overgrowth” with more bony overgrowth representing a worse score
Subchondral Changes	Criteria for “edema-like marrow signal” were removed and simplified into “no intralesional subchondral changes”, “presence of intralesional edema”, or “present of intralesional subchondral cyst”
Cartilage Delamination	Although initially part of the volume filling of cartilage defect domain, given the different mechanism from volume filling delamination given its own domain evaluating the presence or absence of delamination at the chondrolabral junction
Signal Intensity of Repair Tissue	Domain removed entirely given difficulty in assessment with poor-quality MRI

**Fig. 2:**
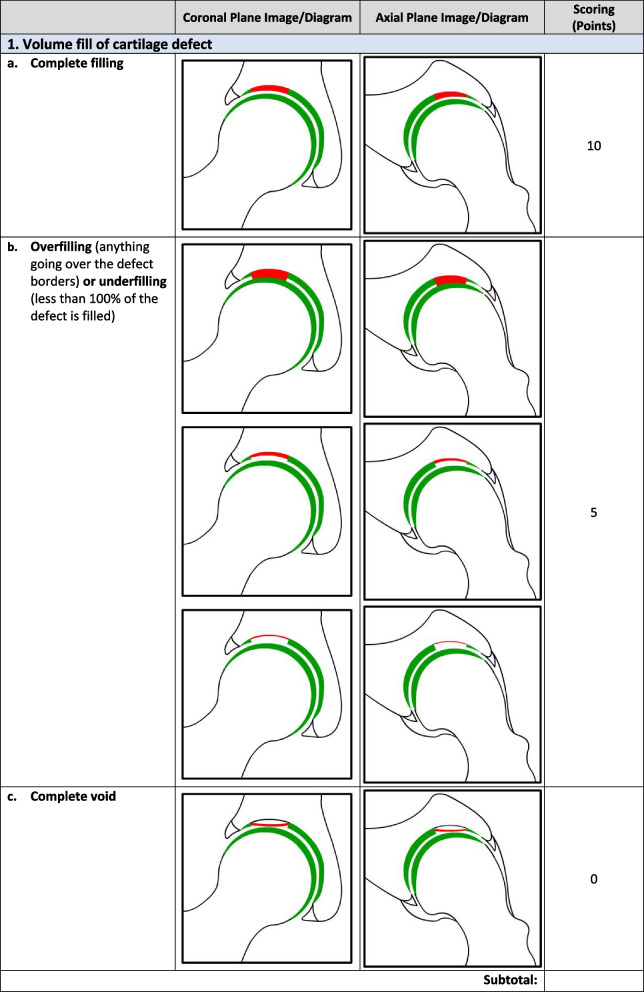

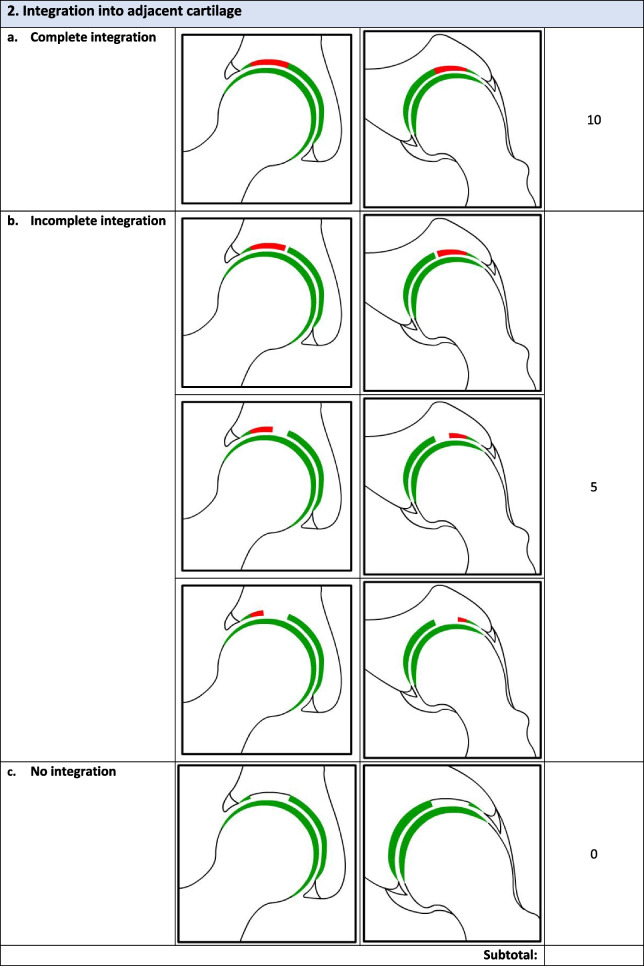

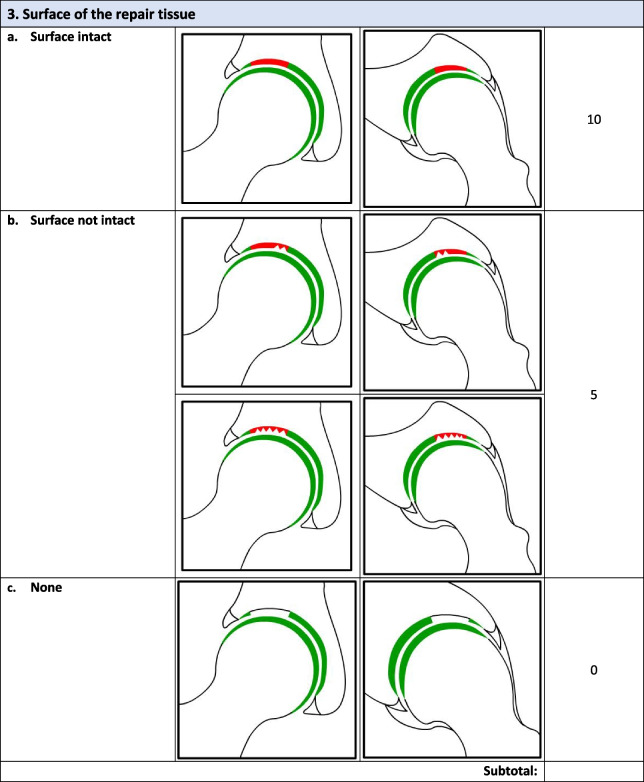

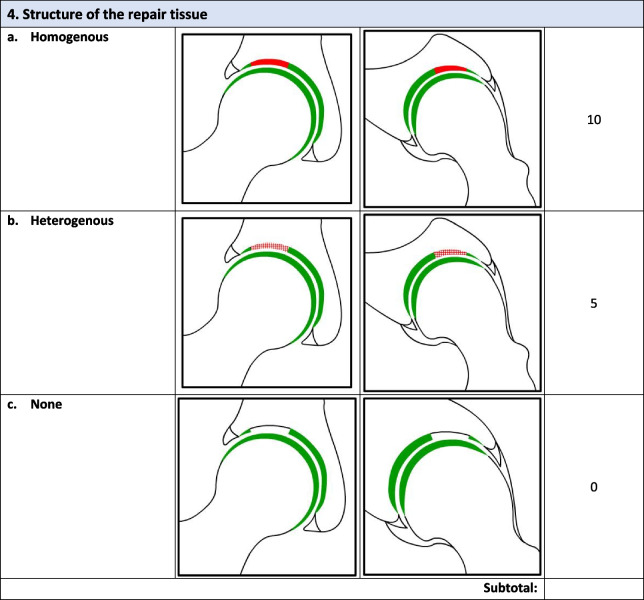

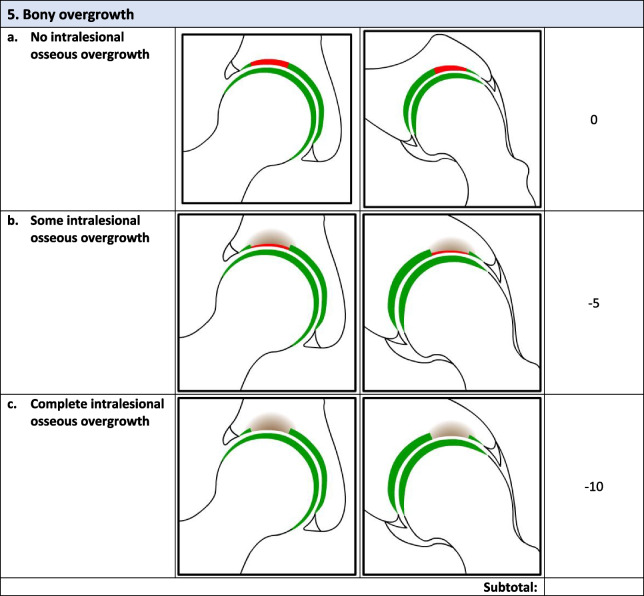

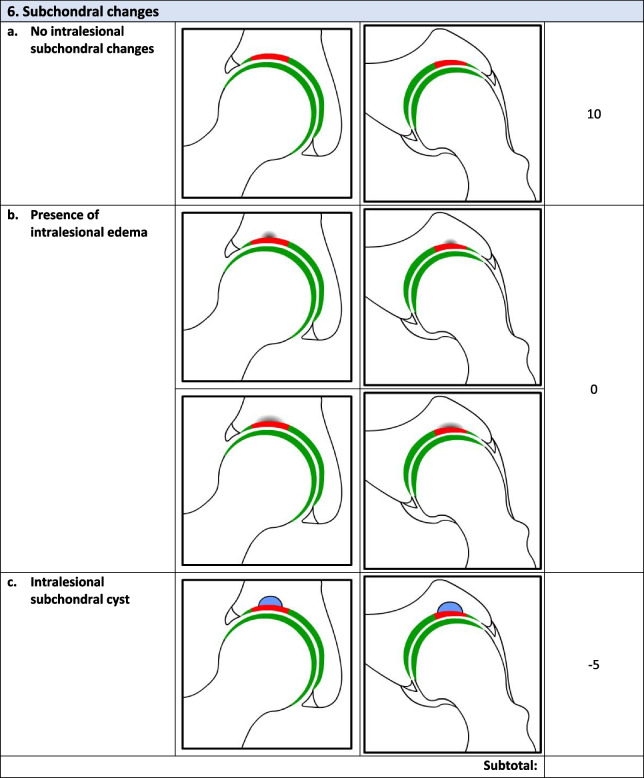

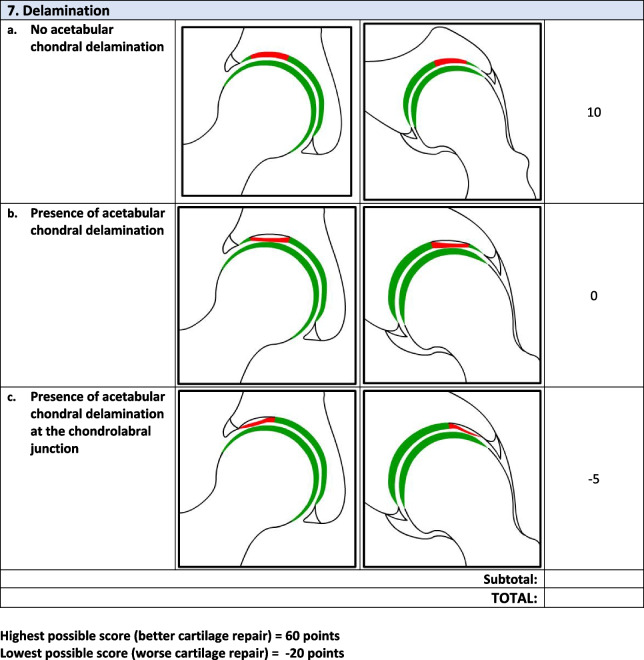
MERCH Score

Following Phase 1 and 2, the final MERCH score was developed with 7 domains, 3 criteria per domain: 1) volume fill of cartilage defect, 2) integration into adjacent cartilage, 3) surface of the repair tissue, 4) structure of the repair tissue, 5) bony overgrowth, 6) subchondral changes, and 7) delamination (Fig. 2). For domains 1 to 4, criteria are scored as 10 (best), 5, and 0 (worst). Where any bony overgrowth has been shown to lead to poor outcomes, domain 5 was scored as 0 (no overgrowth), -5 (some overgrowth), and -10 (complete osseous overgrowth). Subchondral changes and delamination in domains 6 and 7 were scored as 10 (no changes), 0 (presence of edema/delamination), and -5 (presence of a subchondral cyst/delamination at the chondrolabral junction). Therefore, the MERCH score has a highest possible score of 60 points, representing optimal cartilage repair, and a lowest possible score of -20 points, representing the worst possible to no repair. These scores were agreed upon during the consensus meeting based on the severity of the presence or absence of these morphologies in the hip and their impact on cartilage repair.

Participants were also asked about the minimum time post-operatively that they felt they would be able to accurately evaluate cartilage repair on an MRI and 94% (16/17) agreed on 12 months post-operatively.

## Discussion

Overall, in keeping with our hypothesis, this modified Delphi study demonstrated a general consensus on MRI criteria defining post-operative repair and/or reconstruction of hip cartilage. This modified Delphi study established standardized MRI criteria to evaluate hip cartilage repair from pre- to post-operatively across 7 domains including: 1) volume fill of cartilage defect, 2) integration into adjacent cartilage, 3) surface of the repair tissue, 4) structure of the repair tissue, 5) bony overgrowth, 6) subchondral changes and 7) delamination. Participants agreed that these criteria should be evaluated at a minimum of 12 months post-operatively using a 3.0 T MRI.

Throughout the process of this Delphi study, there was a general consensus to simplify the criteria used to evaluate hip cartilage repair. Specifically, the domains evaluating volume filling of the cartilage defect, integration into adjacent cartilage and subchondral changes were all simplified from 4 to 5 criteria with multiple descriptors per domain, to 3 criteria with simple descriptors, while the criteria discussing signal intensity of the repair tissue was removed entirely to help ensure the score would be useful to orthopaedic surgeons and researchers globally with varying access to different MRI technology. Further, it has been shown in the orthopaedic field that utilizing a simpler scoring system with clear terminology results in improved inter- and intra-observer agreement, especially when evaluating an MRI [3, 17, 22]. Furthermore, previous studies discussing cartilage repair in the knee support the notion that use of a 3.0 T MRI at a minimum of 12-months post-operatively is optimal to evaluate cartilage repair and integration [8, 21]. Therefore, it is important that the MERCH score be applied at 12 months or more post-operatively.

As discussed above, the main scoring systems that exist to classify cartilage status in the hip include the SHOMRI and HOAMS scores, which were generally created to evaluate OA in the hip [13, 16, 19]. Previous studies evaluating radiographic outcomes after cartilage repair in the hip used very general terms such as “well-incorporated autograft” or “intact cartilage” [6, 15]. The proposed MERCH scoring system will provide both surgeons and radiologists a structured and universal language which can be used to describe repaired cartilage in the hip post-operatively. Addressing this knowledge gap is critical in our ability to guide patient management and expectations especially in a rapidly evolving field that is seeing an increasing amount of cartilage restoration procedures.

This study was strengthened by the multi-phase, Delphi approach involving both expert surgeons and radiologists across the country. The involvement of both surgeons and radiologists adds to the validity of this study as it encompasses all viewpoints and ensures consistency in both understanding and application of the scoring system by bridging the gap between clinician and radiologist.

There were some limitations to this study. Firstly, given that all physicians who participated were located in Canada, this may limit the generalizability of the results worldwide as different centers may have differential access to high-quality MRI or may have different levels of expertise regarding the ability to read MRI scans. Moreover, inherent to any consensus discussion, it is possible that some participants had strong opinions regarding certain topics which may bias the consensus panel’s decision-making in some areas. We attempted to temper this by offering an initial survey evaluation and anonymous voting during the consensus discussion. Finally, there is potential for bias related to the smaller sample size of 17 participants involved as this may result in less robust conclusions, however this is highly unlikely given the high agreement consistently seen throughout the study.

## Conclusions

This consensus project established a new MRI scoring system to evaluate post-operative cartilage restoration of the hip. The implementation of the MERCH score is essential in our ability to guide patient management and expectations in a rapidly evolving field and will help with standardizing our evaluation of cartilage repair in future research trials. Future directions include performing a validation study of the proposed scoring system to ensure sufficient agreement is present in its application, which the MERCH Investigators have planned. Ultimately, the MERCH score can be used to standardize the evaluation of cartilage repair and guide research trials when evaluating various cartilage restoration procedures in the hip.

### Supplementary Information

**Additional file 1.** 

** Additional file 2.**

## Data Availability

Data sharing is not applicable to this article as no datasets were generated or analysed during the current study.

## Abbreviations

MRI: Magnetic resonance imaging
MERCH: Magnetic Resonance Evaluation of the Repair of Cartilage in the Hip
OA: Osteoarthritis
ICRS: International Cartilage Repair Society
SHOMRI: Scoring Hip Osteoarthritis with Magnetic Resonance Imaging
HOAMS: Hip Osteoarthritis MRI Scoring System
MOCART: Magnetic Resonance Observation of Cartilage Repair Tissue
HiREB: Hamilton Integrated Research Ethics Board
