# Supplementary material for: Defining hip cartilage repair: a modified delphi study to establish the Magnetic Resonance Evaluation of the Repair of Cartilage in the Hip (MERCH) score
Source: J Exp Orthop. 2023 Dec 5;10:129. doi: 10.1186/s40634-023-00676-y (PMC10697921; doi:10.1186/s40634-023-00676-y)
Supplement: Supplementary file 1 — Additional file 1. [file 40634_2023_676_MOESM1_ESM.docx]

Appendix Table 1.

| **1** | **2** | **3** | **4** | **5** | **6** | **7** |
| --- | --- | --- | --- | --- | --- | --- |
| Strongly Disagree | Disagree | Somewhat Disagree | Neutral | Somewhat Agree | Agree | Strongly Agree |
