# Supplementary material for: Defining hip cartilage repair: a modified delphi study to establish the Magnetic Resonance Evaluation of the Repair of Cartilage in the Hip (MERCH) score
Source: J Exp Orthop. 2023 Dec 5;10:129. doi: 10.1186/s40634-023-00676-y (PMC10697921; doi:10.1186/s40634-023-00676-y)
Supplement: Supplementary file 2 — Additional file 2. [file 40634_2023_676_MOESM2_ESM.docx]

# Appendix 2: Phase I Survey Results

**Legend:**

Green text or shading – majority of respondents agreed

Red text or shading – majority of respondents disagreed

*Other comments are presented in this report for general consideration prior to the consensus meeting.

**Demographics of Respondents**

| **N=17** | **No.** | | |
| --- | --- | --- | --- |
| **Annual hip arthroscopy procedures** |  | | |
| Mean (SD) | 75 (50.7) | | |
| Median | 65 | | |
| Maximum | 200 | | |
| Minimum | 10 | | |
|  | **No.** | **(%)** |  |
| **Perform hip arthroscopy procedures?** |  |  |  |
| Yes | 14 | 82.4 |  |
| No | 3 | 17.6 |  |
| **Age** |  |  |  |
| Less than 30 years old | 1 | 5.9 |  |
| 31-40 years old | 7 | 41.2 |  |
| 41-50 years old | 7 | 41.2 |  |
| 51-60 years old | 2 | 11.8 |  |
| **Current position** |  |  |  |
| Orthopaedic surgeon | 11 | 64.7 |  |
| Paediatric orthopaedic surgeon | 4 | 23.5 |  |
| Radiologist | 2 | 11.8 |  |
| **Country of practice** |  |  |  |
| Canada | 17 | 100 |  |
| **Years in Practice** |  |  |  |
| 1-3 years | 2 | 11.8 |  |
| 4-6 years | 5 | 29.4 |  |
| 7-9 years | 4 | 23.5 |  |
| 10-14 years | 3 | 17.6 |  |
| 15-20 years | 2 | 11.8 |  |
| 20+ years | 1 | 5.9 |  |
| **Clinical Setting** |  |  |  |
| Academic Centre Hospital | 14 | 82.3 |  |
| Academic Centre Clinic | 3 | 17.6 |  |
| Community Hospital | 2 | 11.8 |  |
| **Fellowship or Additional Training** |  |  |  |
| Yes | 16 | 94.1 |  |
| No | 1 | 5.9 |  |

# Phase I – Survey re: Proposed MERCH criteria

## MRI PROTOCOL

| **N=17** | **No.** | **(%)** |
| --- | --- | --- |
| **What MRI protocol is recommended for evaluating hip cartilage AT A MINIMUM? Please consider international standards and resource availability.** |  |  |
| 1.5T alone | 2 | 11.8 |
| 1.5T with arthrogram | 7 | 41.2 |
| 3.0T alone | 8 | 47.1 |
| **What plane(s) is/are best/essential for imaging hip cartilage? We understand that the hip is a 3-dimensional structure. However, for our purposes in developing what we hope to be a standardized scoring system, please select the MOST IMPORTANT plane for evaluating ARTICULAR cartilage via MRI.** |  |  |
| Coronal | 5 | 29.4 |
| Sagittal | 2 | 11.8 |
| More than one plane | 10 | 58.8 |
| Coronal + axial oblique | 3 | 30.0 |
| Coronal + sagittal | 2 | 20.0 |
| No planes suggested | 5 | 50.0 |
| **Do you believe that a score to evaluate articular cartilage repair in the hip can reasonably be established using the coronal plane view?** |  |  |
| Yes | 5 | 29.4 |
| Yes, I think the coronal plane view is reasonable to use and will comment on the rest of the survey in this regard,  however, I also feel strongly that additional planes should be incorporated into the score. | 12 | 70.6 |
| **What is the earliest timeframe post-surgery that you believe you will be able to accurately view the first signs of cartilage repair on MRI?** |  |  |
| 0-5 months | 1 | 5.9 |
| 6-12 months | 13 | 76.5 |
| 13-18 months | 3 | 17.6 |
| **At what timepoint post-surgery should MRI imaging occur to evaluate successful cartilage repair and integration?** |  |  |
| 6-12 months | 8 | 47.1 |
| 13-18 months | 8 | 47.1 |
| 18-24 months | 1 | 5.9 |

## Section 1 – VOLUME FILLING OF CARTILAGE DEFECT

| **Volume fill of cartilage defect** | **Image/Diagram** | **Scoring (Points)** |
| --- | --- | --- |
| a. Complete filling OR minor hypertrophy: 100% to 150% filling of total defect volume | 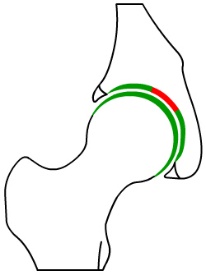 | 20 |
| \| **Strongly**  **disagree** \| **Disagree** \| **Somewhat**  **disagree** \| **Neutral** \| \| **Somewhat**  **agree** \| \| **Agree** \| **Strongly**  **agree** \| \| --- \| --- \| --- \| --- \| --- \| --- \| --- \| --- \| --- \| \|  \|  \|  \| 1 \| \|  \| \| 12 \| 4 \| \| 0 (0%) \| \| \| \| 1 (5.9%) \| \| **16 (94.1%)** \| \| \| \| | \| **Strongly**  **disagree** \| **Disagree** \| **Somewhat**  **disagree** \| **Neutral** \| **Somewhat**  **agree** \| **Agree** \| **Strongly**  **agree** \| \| --- \| --- \| --- \| --- \| --- \| --- \| --- \| \|  \| 1 \| 1 \|  \| 2 \| 9 \| 4 \| \| 2 (11.8%) \| \| \| 0 (0%) \| **15 (88.2%)** \| \| \| |  |
| b. Major hypertrophy ≥150% filling of total defect volume | 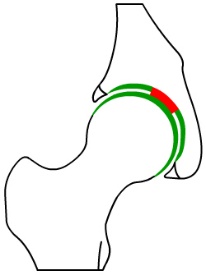 | 15 |
| \| **Strongly**  **disagree** \| **Disagree** \| **Somewhat**  **disagree** \| **Neutral** \| **Somewhat**  **agree** \| **Agree** \| **Strongly**  **agree** \| \| --- \| --- \| --- \| --- \| --- \| --- \| --- \| \|  \| 2 \|  \| 2 \|  \| 10 \| 3 \| \| 2 (11.8%) \| \| \| 2 (11.8%) \| 13 (76.4%) \| \| \| | \| **Strongly**  **disagree** \| **Disagree** \| **Somewhat**  **disagree** \| **Neutral** \| **Somewhat**  **agree** \| **Agree** \| **Strongly**  **agree** \| \| --- \| --- \| --- \| --- \| --- \| --- \| --- \| \|  \| 2 \| 1 \|  \| 4 \| 7 \| 3 \| \| 3 (17.6%) \| \| \| 0 (0%) \| **14 (82.4%)** \| \| \| |  |
| c. Underfilling (>50% of the defect is filled) | 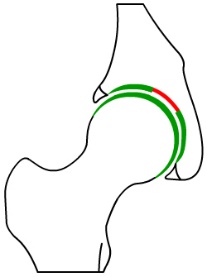 | 10 |
| \| **Strongly**  **disagree** \| **Disagree** \| **Somewhat**  **disagree** \| **Neutral** \| **Somewhat**  **agree** \| **Agree** \| **Strongly**  **agree** \| \| --- \| --- \| --- \| --- \| --- \| --- \| --- \| \|  \|  \|  \|  \|  \| 11 \| 6 \| \| 0 (0%) \| \| \| 0 (0%) \| **17 (100.0%)** \| \| \| | \| **Strongly**  **disagree** \| **Disagree** \| **Somewhat**  **disagree** \| **Neutral** \| **Somewhat**  **agree** \| **Agree** \| **Strongly**  **agree** \| \| --- \| --- \| --- \| --- \| --- \| --- \| --- \| \|  \| 1 \| 2 \|  \| 2 \| 7 \| 5 \| \| 3 (17.6%) \| \| \| 0 (0%) \| **14 (82.4%)** \| \| \| |  |
| d. Severe underfilling (<50% of the defect is filled) | 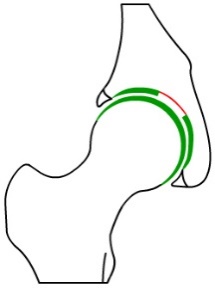 | 5 |
| \| **Strongly**  **disagree** \| **Disagree** \| **Somewhat**  **disagree** \| **Neutral** \| **Somewhat**  **agree** \| **Agree** \| **Strongly**  **agree** \| \| --- \| --- \| --- \| --- \| --- \| --- \| --- \| \|  \|  \|  \| 1 \|  \| 10 \| 6 \| \| 0 (0%) \| \| \| 1 (5.9%) \| **16 (94.1%)** \| \| \| | \| **Strongly**  **disagree** \| **Disagree** \| **Somewhat**  **disagree** \| **Neutral** \| **Somewhat**  **agree** \| **Agree** \| **Strongly**  **agree** \| \| --- \| --- \| --- \| --- \| --- \| --- \| --- \| \|  \| 1 \| 1 \|  \| 2 \| 8 \| 5 \| \| 2 (11.8%) \| \| \| 0 (0%) \| **15 (88.2%)** \| \| \| |  |
| e. Complete delamination | 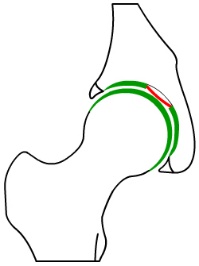 | 0 |
| \| **Strongly**  **disagree** \| **Disagree** \| **Somewhat**  **disagree** \| **Neutral** \| **Somewhat**  **agree** \| **Agree** \| **Strongly**  **agree** \| \| --- \| --- \| --- \| --- \| --- \| --- \| --- \| \|  \|  \|  \| 1 \|  \| 11 \| 5 \| \| 0 (0%) \| \| \| 1 (5.9%) \| **16 (94.1%)** \| \| \| | \| **Strongly**  **disagree** \| **Disagree** \| **Somewhat**  **disagree** \| **Neutral** \| **Somewhat**  **agree** \| **Agree** \| **Strongly**  **agree** \| \| --- \| --- \| --- \| --- \| --- \| --- \| --- \| \|  \| 3 \| 2 \|  \|  \| 8 \| 4 \| \| 5 (29.4%) \| \| \| 0 (0%) \| 12 (70.6%) \| \| \| |  |

**SECTION 1 - COMMENTS (text coded)**

| Images:   - Increase image size (to better show examples of hypertrophy, lack of fill, etc.), zoom in on acetabulum/head - Won’t majority of defects be closer to chondrolabral junction rather than parafoveal? More common location of defect would be more lateral in acetabulum, closer to labrum. |
| --- |
| “Underfilling”, confusion around what this means:   - Is it 50-74% fill or 50-99% fill? (75-99% for hypertrophy)? - Stick with >150% (delete 77-99%) - Should be divided into 25% increments - Complete filling can be 100%, overfilling is anything beyond margins of adjacent cartilage, % is difficult to assess accurately 🡪 use <50% vs >50%, further grading is not accurate for MRI*** |
| “Hypertrophy” is subjective:   - Perhaps only characterize based on filling of total defect volume (not hypertrophy) - Borderline cases will be difficult to assign (e.g. 151% hypertrophy) - Use 125% as cut off for major vs. minor hypertrophy (instead of 150%) - Remove “b”, too difficult to tell (i.e., stick with major vs. minor with certain cut off value) |
| “Delamination”   - Should be provided a score? - Can be a measure of entire cartilage repaired or a percentage of it (e.g. 50% in coronal plane, 25% or 100%) - “Complete delamination” should be reworded as “complete void” - Different mechanism of injury entirely |

**SECTION 1 - PROPOSED REVISIONS BASED ON COMMENTS:**

| **Volume fill of cartilage defect** | **Coronal Plane Image/Diagram** | **Axial Plane Image/Diagram** | **Scoring (Points)** |
| --- | --- | --- | --- |
| 1. Complete filling ~~OR minor hypertrophy: 100% to 150% filling of total defect volume~~ | 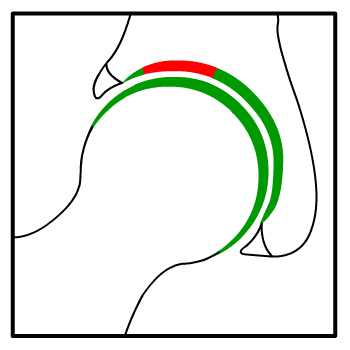 | 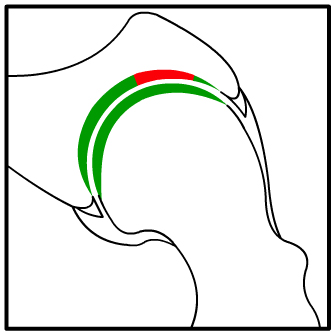 | 20 |
| 1. Overfilling (anything going over defect borders) ~~Major hypertrophy ≥150% filling of total defect volume~~ | 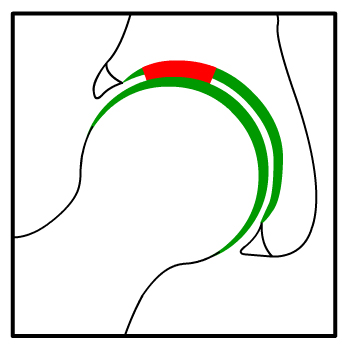 | 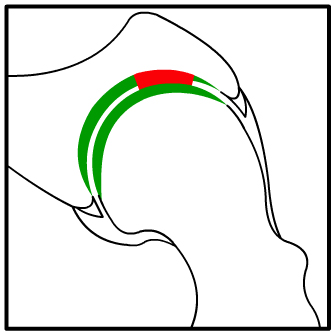 | 15 |
| 1. Underfilling (>50% of the defect is filled) | 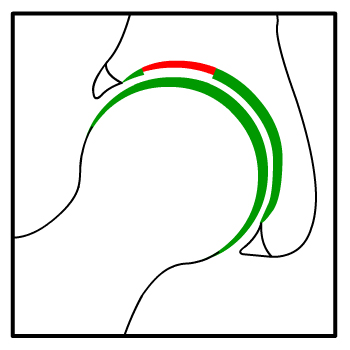 | 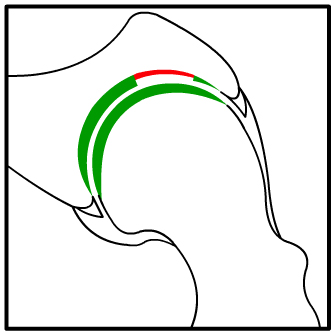 | 10 |
| 1. Severe underfilling (<50% of the defect is filled) | 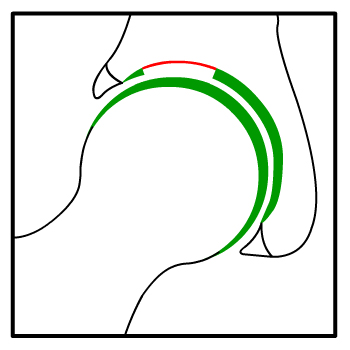 | 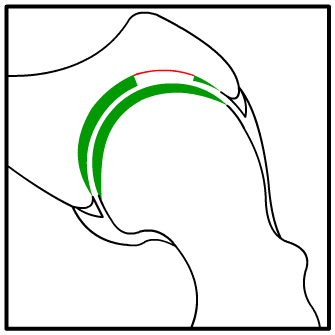 | 5 |
| 1. Complete ~~delamination~~ void | 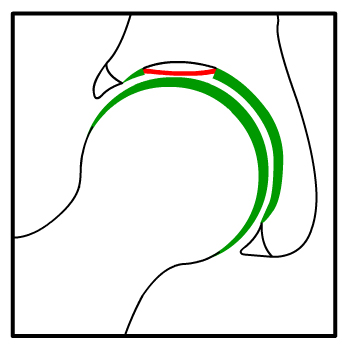 | 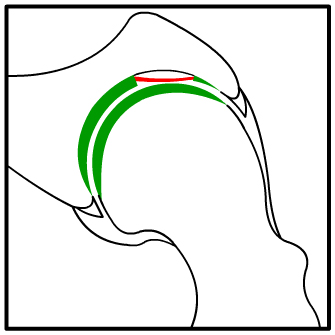 | 0 |

**SECTION 1 - CONSENSUS MEETING QUESTION(S):**

1. Vote on changes above.

## Section 2 – INTEGRATION INTO ADJACENT CARTILAGE

| **Integration into adjacent cartilage** | **Image/Diagram** | **Scoring (Points)** |
| --- | --- | --- |
| a. Complete integration | 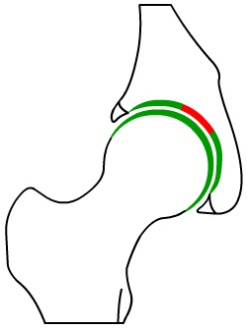 | 15 |
| \| **Strongly**  **disagree** \| **Disagree** \| **Somewhat**  **disagree** \| **Neutral** \| **Somewhat**  **agree** \| **Agree** \| **Strongly**  **agree** \| \| --- \| --- \| --- \| --- \| --- \| --- \| --- \| \|  \|  \|  \|  \| 2 \| 8 \| 7 \| \| 0 (0%) \| \| \| 0 (0%) \| **17 (100.0%)** \| \| \| | \| **Strongly**  **disagree** \| **Disagree** \| **Somewhat**  **disagree** \| **Neutral** \| **Somewhat**  **agree** \| **Agree** \| **Strongly**  **agree** \| \| --- \| --- \| --- \| --- \| --- \| --- \| --- \| \|  \|  \|  \|  \| 1 \| 11 \| 5 \| \| 0 (0%) \| \| \| 0 (0%) \| **17 (100.0%)** \| \| \| |  |
| b. Split-like defect at repair tissue and native cartilage interface | 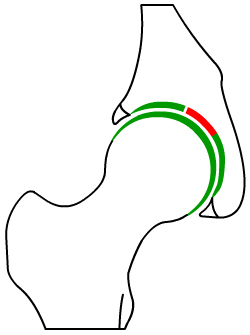 | 10 |
| \| **Strongly**  **disagree** \| **Disagree** \| **Somewhat**  **disagree** \| **Neutral** \| **Somewhat**  **agree** \| **Agree** \| **Strongly**  **agree** \| \| --- \| --- \| --- \| --- \| --- \| --- \| --- \| \|  \|  \|  \|  \| 2 \| 9 \| 6 \| \| 0 (0%) \| \| \| 0 (0%) \| **17 (100.0%)** \| \| \| | \| **Strongly**  **disagree** \| **Disagree** \| **Somewhat**  **disagree** \| **Neutral** \| **Somewhat**  **agree** \| **Agree** \| **Strongly**  **agree** \| \| --- \| --- \| --- \| --- \| --- \| --- \| --- \| \|  \|  \|  \|  \| 1 \| 12 \| 4 \| \| 0 (0%) \| \| \| 0 (0%) \| **17 (100.0%)** \| \| \| |  |
| c. Defect at repair tissue and native cartilage interface <50% of repair tissue length | 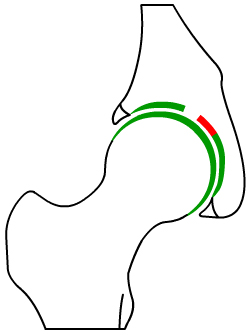 | 5 |
| \| **Strongly**  **disagree** \| **Disagree** \| **Somewhat**  **disagree** \| **Neutral** \| **Somewhat**  **agree** \| **Agree** \| **Strongly**  **agree** \| \| --- \| --- \| --- \| --- \| --- \| --- \| --- \| \|  \|  \|  \|  \| 1 \| 12 \| 4 \| \| 0 (0%) \| \| \| 0 (0%) \| **17 (100.0%)** \| \| \| | \| **Strongly**  **disagree** \| **Disagree** \| **Somewhat**  **disagree** \| **Neutral** \| **Somewhat**  **agree** \| **Agree** \| **Strongly**  **agree** \| \| --- \| --- \| --- \| --- \| --- \| --- \| --- \| \|  \|  \|  \|  \| 3 \| 10 \| 4 \| \| 0 (0%) \| \| \| 0 (0%) \| **17 (100.0%)** \| \| \| |  |
| d. Defect at repair tissue and native cartilage interface ≥50% of repair tissue length | 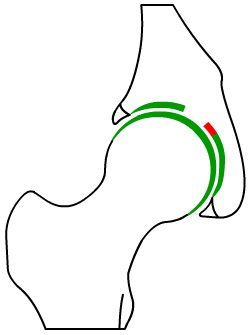 | 0 |
| \| **Strongly**  **disagree** \| **Disagree** \| **Somewhat**  **disagree** \| **Neutral** \| **Somewhat**  **agree** \| **Agree** \| **Strongly**  **agree** \| \| --- \| --- \| --- \| --- \| --- \| --- \| --- \| \|  \|  \|  \|  \| 1 \| 12 \| 4 \| \| 0 (0%) \| \| \| 0 (0%) \| **17 (100.0%)** \| \| \| | \| **Strongly**  **disagree** \| **Disagree** \| **Somewhat**  **disagree** \| **Neutral** \| **Somewhat**  **agree** \| **Agree** \| **Strongly**  **agree** \| \| --- \| --- \| --- \| --- \| --- \| --- \| --- \| \|  \|  \|  \|  \| 2 \| 11 \| 4 \| \| 0 (0%) \| \| \| 0 (0%) \| **17 (100.0%)** \| \| \| |  |

**SECTION 2 - COMMENTS (text coded)**

| - For C and D - what if there is delamination or increased signal under repair tissue meeting <50% or >50% - would these fall into c/d, or because there is no defect' do they fall into B? |
| --- |
| - b. Split like defect should be based on less than or equal to 2mm*** |
| - More than one plane view needed |
| - Should use 25%, 50%, and 100% in coronal plane (optimal) |
| - Integration difficult to assess, either integrated or not |

**SECTION 2 - PROPOSED REVISIONS BASED ON COMMENTS:**

| **Integration into adjacent cartilage** | **Coronal Plane Image/Diagram** | **Axial Plane Image/Diagram** | **Scoring (Points)** |
| --- | --- | --- | --- |
| 1. Complete integration | 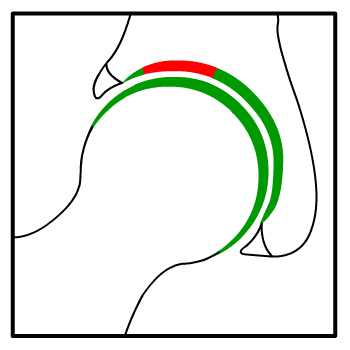 | 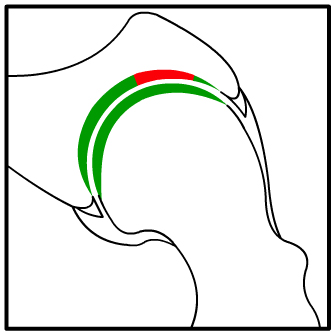 | 15 |
| 1. Split-like defect at repair tissue and native cartilage interface is ≤ 2mm | 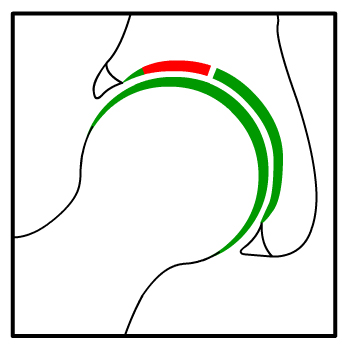 | 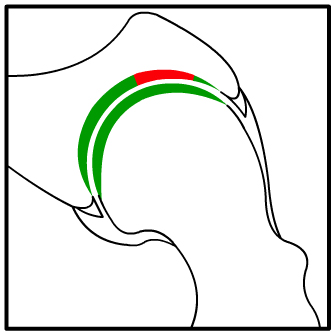 | 10 |
| 1. Defect at repair tissue and native cartilage interface is <50% of repair tissue length | 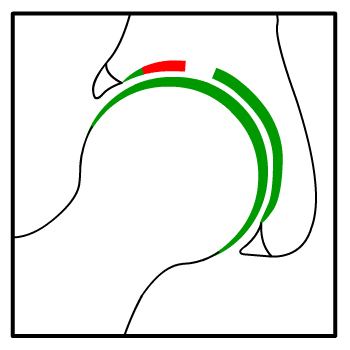 | 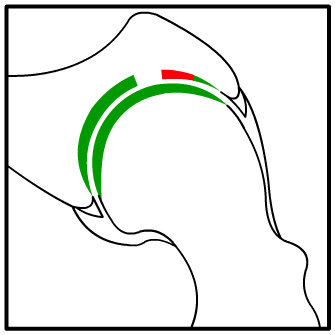 | 5 |
| 1. Defect at repair tissue and native cartilage interface is ≥50% of repair tissue length | 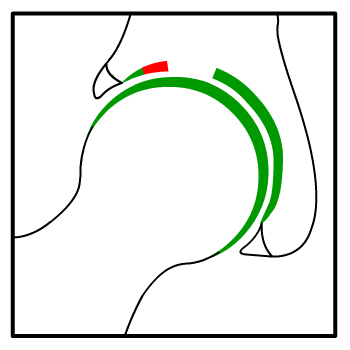 | 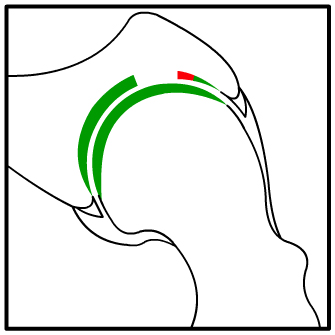 | 0 |

**SECTION 2 – CONSENSUS MEETING QUESTION(S):**

1. Do we keep divisions shown in c and d, or simplify to “any defect”?
2. Do we simplify further to ‘complete integration’ vs. ‘incomplete integration’?
3. Vote on changes above.

## Section 3 – SURFACE OF THE REPAIR TISSUE

| **Surface of the repair tissue** | **Image/Diagram** | **Scoring (Points)** |
| --- | --- | --- |
| a. Surface intact | 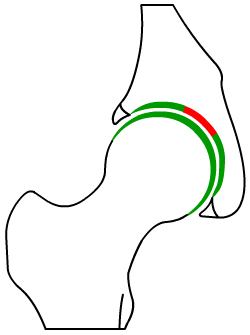 | 10 |
| \| **Strongly**  **disagree** \| **Disagree** \| **Somewhat**  **disagree** \| **Neutral** \| \| **Somewhat**  **agree** \| \| **Agree** \| **Strongly**  **agree** \| \| --- \| --- \| --- \| --- \| --- \| --- \| --- \| --- \| --- \| \|  \|  \| 1 \|  \| \|  \| \| 10 \| 6 \| \| 1 (5.9%) \| \| \| \| 0 (0%) \| \| **16 (94.1%)** \| \| \| \| | \| **Strongly**  **disagree** \| **Disagree** \| **Somewhat**  **disagree** \| **Neutral** \| \| **Somewhat**  **agree** \| \| **Agree** \| **Strongly**  **agree** \| \| --- \| --- \| --- \| --- \| --- \| --- \| --- \| --- \| --- \| \|  \|  \| 2 \| 1 \| \|  \| \| 9 \| 5 \| \| 2 (11.8%) \| \| \| \| 1 (5.9%) \| \| **14 (82.4%)** \| \| \| \| |  |
| b. Surface irregular <50% of repair tissue diameter | 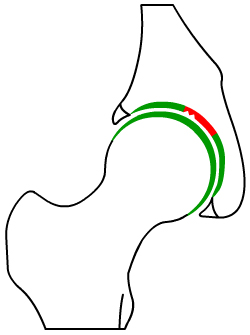 | 5 |
| \| **Strongly**  **disagree** \| **Disagree** \| **Somewhat**  **disagree** \| **Neutral** \| \| **Somewhat**  **agree** \| \| **Agree** \| **Strongly**  **agree** \| \| --- \| --- \| --- \| --- \| --- \| --- \| --- \| --- \| --- \| \|  \|  \| 1 \|  \| \|  \| \| 10 \| 6 \| \| 1 (5.9%) \| \| \| \| 0 (0%) \| \| **16 (94.1%)** \| \| \| \| | \| **Strongly**  **disagree** \| **Disagree** \| **Somewhat**  **disagree** \| **Neutral** \| \| **Somewhat**  **agree** \| \| **Agree** \| **Strongly**  **agree** \| \| --- \| --- \| --- \| --- \| --- \| --- \| --- \| --- \| --- \| \|  \|  \| 2 \| 1 \| \| 2 \| \| 7 \| 5 \| \| 2 (11.8%) \| \| \| \| 1 (5.9%) \| \| **14 (82.4%)** \| \| \| \| |  |
| c. Surface irregular ≥50% of repair tissue diameter | 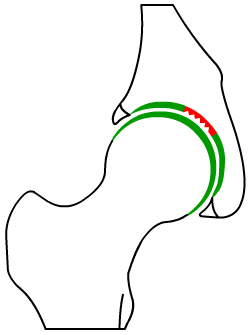 | 0 |
| \| **Strongly**  **disagree** \| **Disagree** \| **Somewhat**  **disagree** \| **Neutral** \| **Somewhat**  **agree** \| **Agree** \| **Strongly**  **agree** \| \| --- \| --- \| --- \| --- \| --- \| --- \| --- \| \|  \|  \| 1 \|  \|  \| 11 \| 5 \| \| 1 (5.9%) \| \| \| 0 (0%) \| **16 (94.1%)** \| \| \| | \| **Strongly**  **disagree** \| **Disagree** \| **Somewhat**  **disagree** \| **Neutral** \| \| **Somewhat**  **agree** \| **Agree** \| **Strongly**  **agree** \| \| \| --- \| --- \| --- \| --- \| --- \| --- \| --- \| --- \| --- \| \|  \|  \| 2 \| 1 \| \| 1 \| 8 \| 5 \| \| \| 2 (11.8%) \| \| \| 1 (5.9%) \| **14 (82.4%)** \| \| \| \| |  |

**SECTION 3 – COMMENTS (text coded)**

| Image:   - Increase the size and detail of the images to better exemplify ‘irregularity’ to 50% - Surface irregularity is typically seen at arthroscopy, not clear if MRI is at the stage where it can truly delineate what the surfaces look like. Diagram should highlight surface detail, perhaps not using a hip diagram and simply showing what intact and non intact images look like. |
| --- |
| “Irregular”:   - Simplify to 2 categories: regular vs. irregular |

**SECTION 3 – PROPOSED REVISIONS BASED ON COMMENTS:**

| **Surface of the repair tissue** | **Coronal Plane Image/Diagram** | **Axial Plane Image/Diagram** | **Scoring (Points)** |
| --- | --- | --- | --- |
| 1. Surface intact | 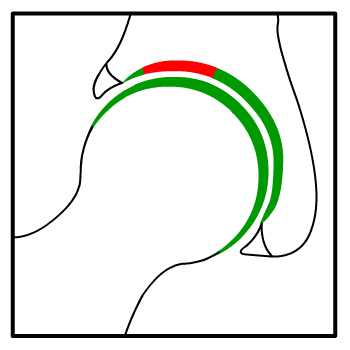 | 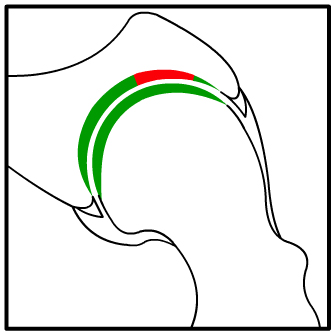 | ~~10~~  15 |
| 1. Surface irregular <50% of repair tissue diameter | 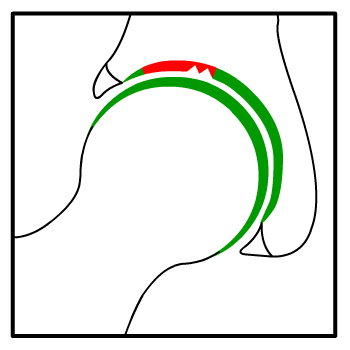 | 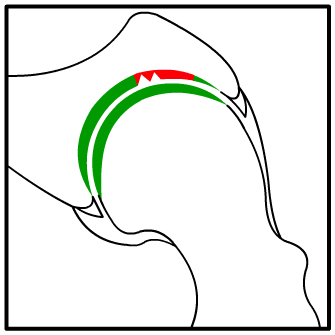 | 5 |
| 1. Surface irregular ≥50% of repair tissue diameter | 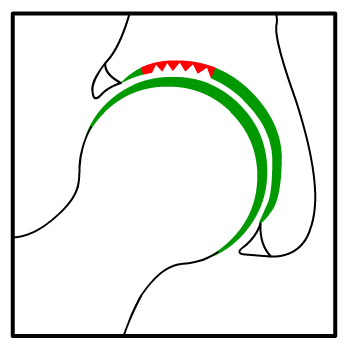 | 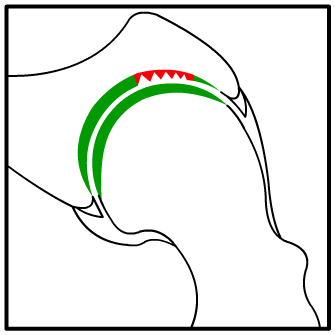 | 0 |

**SECTION 3 – CONSENSUS MEETING QUESTION(S):**

1. Do we simplify to ‘regular’ vs. ‘irregular’?
2. Vote on changes above.

## Section 4 – STRUCTURE OF THE REPAIR TISSUE

| **Structure of the repair tissue** | **Image/Diagram** | **Scoring (Points)** |
| --- | --- | --- |
| a. Homogenous | 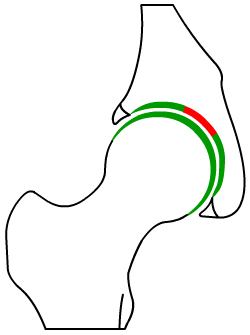 | 10 |
| \| **Strongly**  **disagree** \| **Disagree** \| **Somewhat**  **disagree** \| **Neutral** \| **Somewhat**  **agree** \| **Agree** \| **Strongly**  **agree** \| \| --- \| --- \| --- \| --- \| --- \| --- \| --- \| \|  \|  \|  \|  \| 4 \| 10 \| 3 \| \| 0 (0%) \| \| \| 0 (0%) \| **17 (100.0%)** \| \| \| | \| **Strongly**  **disagree** \| **Disagree** \| **Somewhat**  **disagree** \| **Neutral** \| **Somewhat**  **agree** \| **Agree** \| **Strongly**  **agree** \| \| --- \| --- \| --- \| --- \| --- \| --- \| --- \| \|  \|  \|  \| 1 \| 4 \| 8 \| 4 \| \| 0 (0%) \| \| \| 1 (5.9%) \| **16 (94.1%)** \| \| \| |  |
| b. Heterogenous | 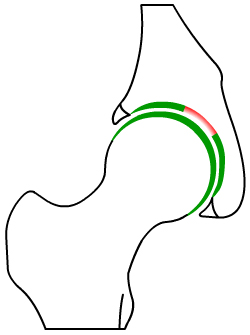 | 0 |
| \| **Strongly**  **disagree** \| **Disagree** \| **Somewhat**  **disagree** \| **Neutral** \| **Somewhat**  **agree** \| **Agree** \| **Strongly**  **agree** \| \| --- \| --- \| --- \| --- \| --- \| --- \| --- \| \|  \|  \|  \|  \| 3 \| 12 \| 2 \| \| 0 (0%) \| \| \| 0 (0%) \| **17 (100.0%)** \| \| \| | \| **Strongly**  **disagree** \| **Disagree** \| **Somewhat**  **disagree** \| **Neutral** \| **Somewhat**  **agree** \| **Agree** \| **Strongly**  **agree** \| \| --- \| --- \| --- \| --- \| --- \| --- \| --- \| \|  \|  \|  \| 1 \| 5 \| 8 \| 3 \| \| 0 (0%) \| \| \| 1 (5.9%) \| **16 (94.1%)** \| \| \| |  |

**SECTION 4 – COMMENTS (text coded)**

| Heterogeneous vs homogeneous   - How defined? More description? - Heterogenous image can be closely dotted or different colour rather than thin line |
| --- |

**SECTION 4 – PROPOSED REVISIONS BASED ON COMMENTS:**

| **Structure of the repair tissue** | **Coronal Plane Image/Diagram** | **Axial Plane Image/Diagram** | **Scoring (Points)** |
| --- | --- | --- | --- |
| 1. Homogenous | 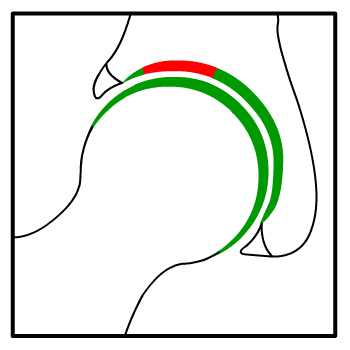 | 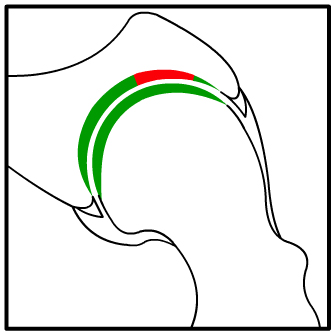 | ~~10~~  15 |
| 1. Heterogenous | 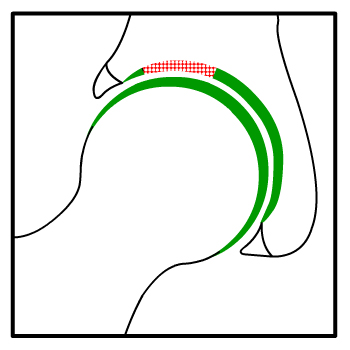 | 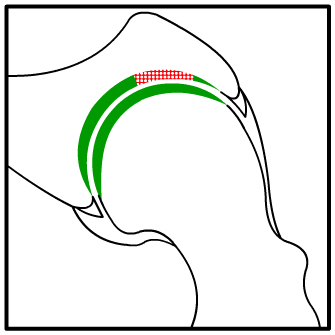 | 0 |

**SECTION 4 – CONSENSUS MEETING QUESTION(S):**

1. Is this the best terminology? Should ‘consistent’ and ‘inconsistent’ be used instead?
2. Vote on changes above.

## Section 5 – SIGNAL INTENSITY OF THE REPAIR TISSUE

| **Signal intensity of the repair tissue** | **Image/Diagram** | **Scoring (Points)** |
| --- | --- | --- |
| a. Normal | 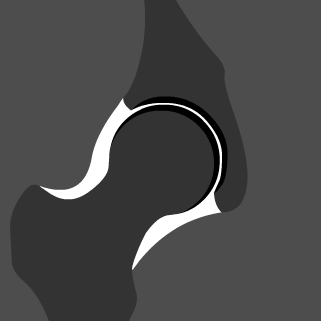 | 15 |
| \| **Strongly**  **disagree** \| **Disagree** \| **Somewhat**  **disagree** \| **Neutral** \| \| **Somewhat**  **agree** \| **Agree** \| **Strongly**  **agree** \| \| \| --- \| --- \| --- \| --- \| --- \| --- \| --- \| --- \| --- \| \|  \|  \|  \|  \| \| 3 \| 8 \| 6 \| \| \| 0 (0%) \| \| \| 0 (0%) \| **17 (100.0%)** \| \| \| \| | \| **Strongly**  **disagree** \| **Disagree** \| **Somewhat**  **disagree** \| **Neutral** \| \| **Somewhat**  **agree** \| **Agree** \| **Strongly**  **agree** \| \| \| --- \| --- \| --- \| --- \| --- \| --- \| --- \| --- \| --- \| \|  \|  \|  \|  \| \| 2 \| 10 \| 5 \| \| \| 0 (0%) \| \| \| 0 (0%) \| **17 (100.0%)** \| \| \| \| |  |
| b. Minor abnormal-minor hyperintense OR minor hypointense | 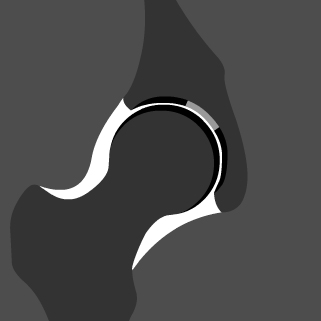 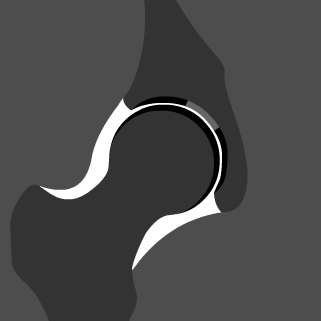 | 10 |
| \| **Strongly**  **disagree** \| **Disagree** \| **Somewhat**  **disagree** \| **Neutral** \| \| **Somewhat**  **agree** \| **Agree** \| **Strongly**  **agree** \| \| \| --- \| --- \| --- \| --- \| --- \| --- \| --- \| --- \| --- \| \|  \|  \|  \|  \| \| 4 \| 7 \| 6 \| \| \| 0 (0%) \| \| \| 0 (0%) \| **17 (100.0%)** \| \| \| \| | \| **Strongly**  **disagree** \| **Disagree** \| **Somewhat**  **disagree** \| **Neutral** \| \| **Somewhat**  **agree** \| **Agree** \| **Strongly**  **agree** \| \| \| --- \| --- \| --- \| --- \| --- \| --- \| --- \| --- \| --- \| \|  \|  \|  \|  \| \| 5 \| 9 \| 3 \| \| \| 0 (0%) \| \| \| 0 (0%) \| **17 (100.0%)** \| \| \| \| |  |
| c. Severely abnormal-almost fluid like OR close to subchondral plate signal | 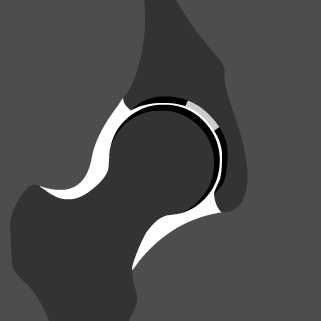 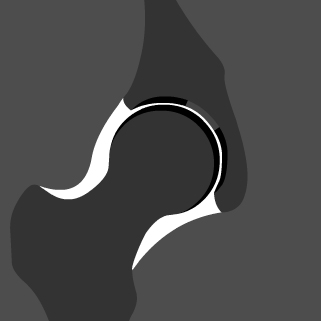 | 5 |
| \| **Strongly**  **disagree** \| **Disagree** \| **Somewhat**  **disagree** \| **Neutral** \| \| **Somewhat**  **agree** \| **Agree** \| **Strongly**  **agree** \| \| \| --- \| --- \| --- \| --- \| --- \| --- \| --- \| --- \| --- \| \|  \|  \|  \|  \| \| 3 \| 8 \| 6 \| \| \| 0 (0%) \| \| \| 0 (0%) \| **17 (100.0%)** \| \| \| \| | \| **Strongly**  **disagree** \| **Disagree** \| **Somewhat**  **disagree** \| **Neutral** \| \| **Somewhat**  **agree** \| **Agree** \| **Strongly**  **agree** \| \| \| --- \| --- \| --- \| --- \| --- \| --- \| --- \| --- \| --- \| \|  \|  \|  \|  \| \| 4 \| 10 \| 3 \| \| \| 0 (0%) \| \| \| 0 (0%) \| **17 (100.0%)** \| \| \| \| |  |

**SECTION 5 – COMMENTS (text coded)**

| b.   - Add: involving less than 50% of repair tissue volume or diameter - Remove right side image (confusing and will be unable to differentiate on MRI) - Should be given same score as c |
| --- |
| c.   - Add: involving greater than 50% of volume or diameter - Remove right side image (confusing and will be unable to differentiate on MRI) - Should be given same score as b |
| Images:   - Zoom in, add colour to help differentiate - Consistent granularity is difficult across sites, should make more basic/clear |

**SECTION 5 – PROPOSED REVISIONS BASED ON COMMENTS:**

| **Signal intensity of the repair tissue** | **Coronal Plane Image/Diagram** | **Axial Plane Image/Diagram** | **Scoring (Points)** |
| --- | --- | --- | --- |
| 1. Normal | 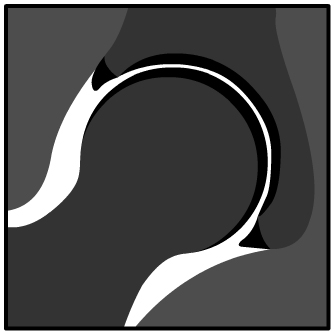 | 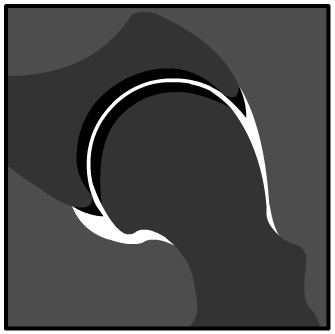 | 15 |
| 1. Minor abnormal-minor hyperintense OR minor hypointense | 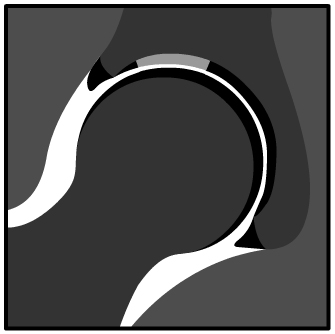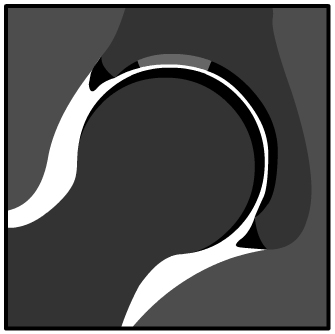 | 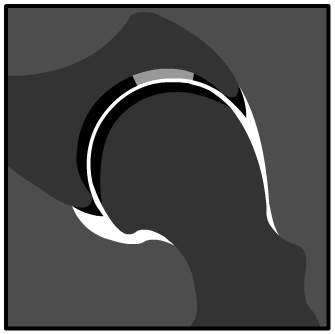  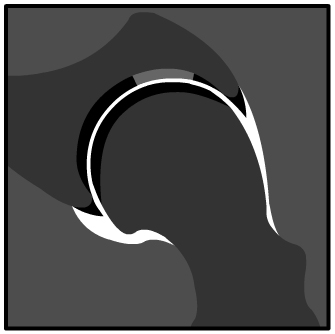 | 10 |
| 1. Severely abnormal-almost fluid like OR close to subchondral plate signal | 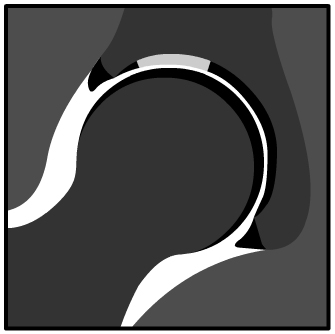  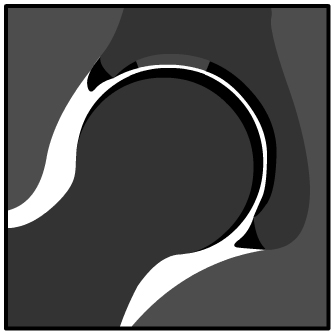 | 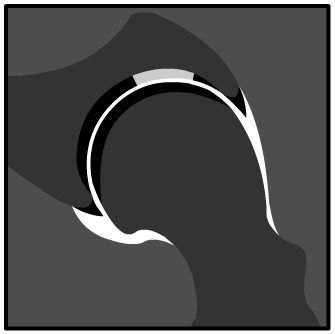  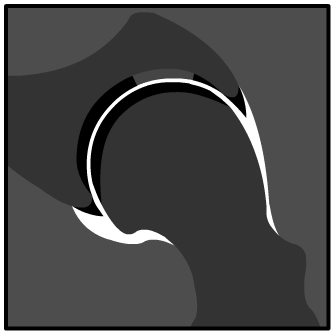 | ~~5~~  0 |

**SECTION 5 -CONSENSUS MEETING QUESTION(S):**

1. ***Should these criteria be removed entirely? Are these evaluations possible with a poor-quality MRI?
2. Alternatively, should the criteria be more specific (e.g., <50% vs. >50%)?
3. Vote on changes above, or to remove entirely.

## Section 6 – BONY DEFECT OR BONY OVERGROWTH

| **Bony defect or bony overgrowth** | **Image/Diagram** | **Scoring (Points)** |
| --- | --- | --- |
| a. No bony defect or bony overgrowth | 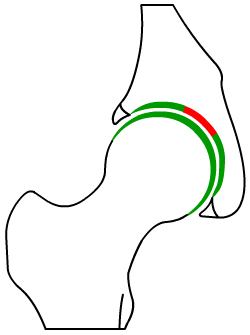 | 10 |
| \| **Strongly**  **disagree** \| **Disagree** \| **Somewhat**  **disagree** \| **Neutral** \| \| **Somewhat**  **agree** \| **Agree** \| **Strongly**  **agree** \| \| \| --- \| --- \| --- \| --- \| --- \| --- \| --- \| --- \| --- \| \|  \|  \| 1 \|  \| \|  \| 10 \| 6 \| \| \| 1 (5.9%) \| \| \| 0 (0%) \| **16 (94.1%)** \| \| \| \| | \| **Strongly**  **disagree** \| **Disagree** \| **Somewhat**  **disagree** \| **Neutral** \| \| **Somewhat**  **agree** \| **Agree** \| **Strongly**  **agree** \| \| \| --- \| --- \| --- \| --- \| --- \| --- \| --- \| --- \| --- \| \|  \|  \|  \| 1 \| \|  \| 11 \| 5 \| \| \| 0 (0%) \| \| \| 1 (5.9%) \| **16 (94.1%)** \| \| \| \| |  |
| b. Bony defect: depth < thickness of adjacent cartilage OR overgrowth <50% of adjacent cartilage | 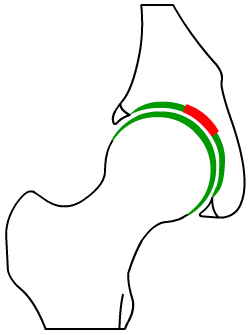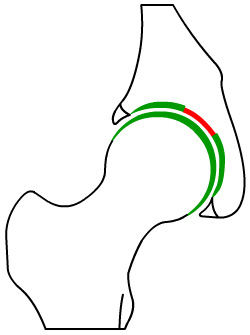 | 5 |
| \| **Strongly**  **disagree** \| **Disagree** \| **Somewhat**  **disagree** \| **Neutral** \| \| **Somewhat**  **agree** \| **Agree** \| **Strongly**  **agree** \| \| \| --- \| --- \| --- \| --- \| --- \| --- \| --- \| --- \| --- \| \|  \|  \| 1 \|  \| \| 1 \| 13 \| 2 \| \| \| 1 (5.9%) \| \| \| 0 (0%) \| **16 (94.1%)** \| \| \| \| | \| **Strongly**  **disagree** \| **Disagree** \| **Somewhat**  **disagree** \| **Neutral** \| \| **Somewhat**  **agree** \| **Agree** \| **Strongly**  **agree** \| \| \| --- \| --- \| --- \| --- \| --- \| --- \| --- \| --- \| --- \| \|  \|  \|  \| 1 \| \| 1 \| 10 \| 5 \| \| \| 0 (0%) \| \| \| 1 (5.9%) \| **16 (94.1%)** \| \| \| \| |  |
| c. Bony defect: depth ≥ thickness of adjacent cartilage OR overgrowth ≥50% of adjacent cartilage | 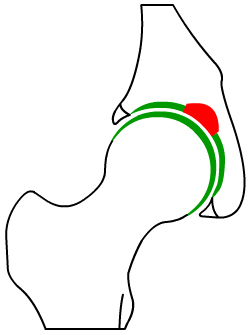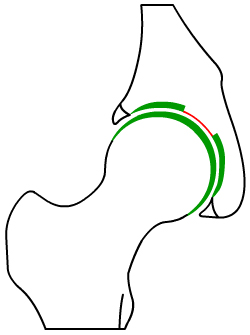 | 0 |
| \| **Strongly**  **disagree** \| **Disagree** \| **Somewhat**  **disagree** \| **Neutral** \| \| **Somewhat**  **agree** \| **Agree** \| **Strongly**  **agree** \| \| \| --- \| --- \| --- \| --- \| --- \| --- \| --- \| --- \| --- \| \|  \|  \| 1 \|  \| \| 2 \| 10 \| 4 \| \| \| 1 (5.9%) \| \| \| 0 (0%) \| **16 (94.1%)** \| \| \| \| | \| **Strongly**  **disagree** \| **Disagree** \| **Somewhat**  **disagree** \| **Neutral** \| \| **Somewhat**  **agree** \| **Agree** \| **Strongly**  **agree** \| \| \| --- \| --- \| --- \| --- \| --- \| --- \| --- \| --- \| --- \| \|  \|  \|  \| 1 \| \| 1 \| 9 \| 6 \| \| \| 0 (0%) \| \| \| 1 (5.9%) \| **16 (94.1%)** \| \| \| \| |  |

**SECTION 6 - COMMENTS (text coded)**

| “Bone overgrowth”   - Should be put in own category - Defect width <50% vs 100% - Cut off should be 25% |
| --- |
| Separate bony defect and bony overgrowth into different categories   - May use scoring of 0, -5, -10 because more bone growth is bad (compared to cartilage) |
| Should focus on cartilage presence or absence |

**SECTION 6 - PROPOSED REVISIONS BASED ON COMMENTS:**

| **Bony defect or bony overgrowth** | **Coronal Plane Image/Diagram** | **Axial Plane Image/Diagram** | **Scoring (Points)** |
| --- | --- | --- | --- |
| 1. No bony defect o~~r bony overgrowth~~ | 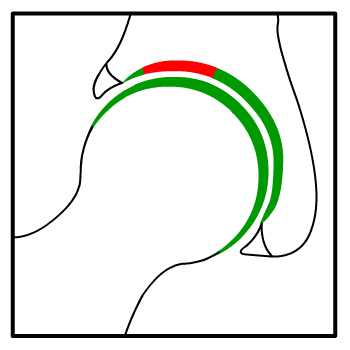 | 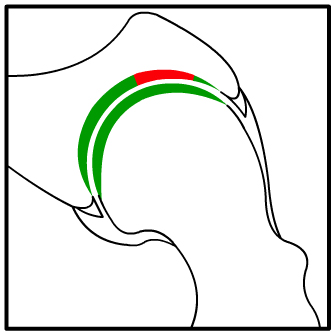 | ~~10~~  0 |
| 1. Bony defect: < 2mm ~~overgrowth <50% of adjacent cartilage~~ | 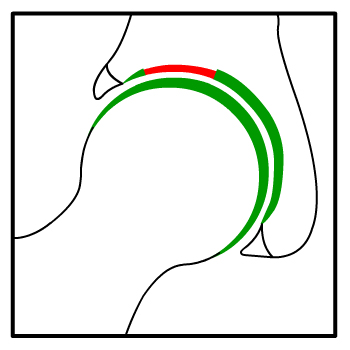 | 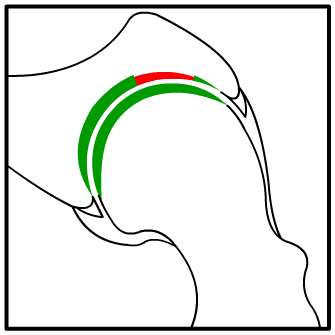 | ~~5~~  -5 |
| 1. Bony defect: ≥ 2mm ~~overgrowth ≥50% of adjacent cartilage~~ | 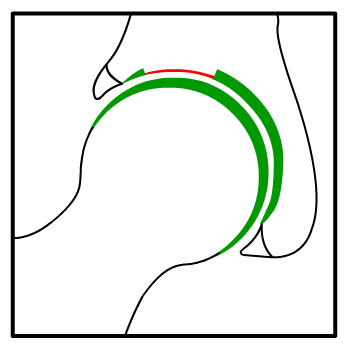 | 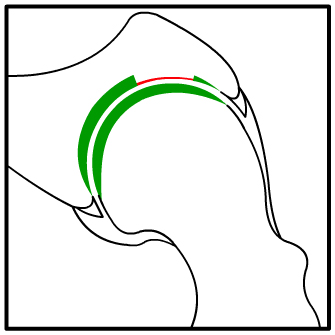 | ~~0~~  -10 |

**SECTION 6 - CONSENSUS MEETING QUESTION(S):**

1. Simplify criteria to ‘presence of bone: yes/no’?
2. If presence of bone, should score remove points (e.g., -5 points)?
3. If no presence of bone, should score be 0?
4. Vote on changes above.

## Section 7 – SUBCHONDRAL CHANGES

| **Subchondral changes** | **Image/Diagram** | **Scoring (Points)** |
| --- | --- | --- |
| a. No major subchondral changes | 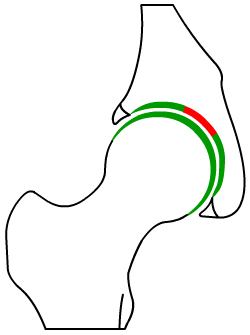 | 20 |
| \| **Strongly**  **disagree** \| **Disagree** \| **Somewhat**  **disagree** \| **Neutral** \| \| **Somewhat**  **agree** \| **Agree** \| **Strongly**  **agree** \| \| \| --- \| --- \| --- \| --- \| --- \| --- \| --- \| --- \| --- \| \|  \|  \|  \|  \| \|  \| 11 \| 6 \| \| \| 0 (0%) \| \| \| 0 (0%) \| **17 (100.0%)** \| \| \| \| | \| **Strongly**  **disagree** \| **Disagree** \| **Somewhat**  **disagree** \| **Neutral** \| \| **Somewhat**  **agree** \| **Agree** \| **Strongly**  **agree** \| \| \| --- \| --- \| --- \| --- \| --- \| --- \| --- \| --- \| --- \| \|  \|  \|  \|  \| \| 1 \| 10 \| 6 \| \| \| 0 (0%) \| \| \| 0 (0%) \| **17 (100.0%)** \| \| \| \| |  |
| b. Minor edema-like marrow signal – maximum diameter <50% of repair tissue diameter | 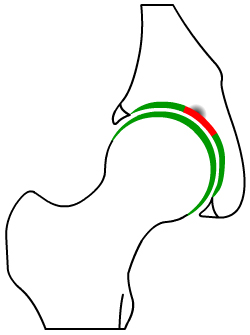 | 15 |
| \| **Strongly**  **disagree** \| **Disagree** \| **Somewhat**  **disagree** \| **Neutral** \| \| **Somewhat**  **agree** \| **Agree** \| **Strongly**  **agree** \| \| \| --- \| --- \| --- \| --- \| --- \| --- \| --- \| --- \| --- \| \|  \|  \|  \|  \| \| 1 \| 11 \| 5 \| \| \| 0 (0%) \| \| \| 0 (0%) \| **17 (100.0%)** \| \| \| \| | \| **Strongly**  **disagree** \| **Disagree** \| **Somewhat**  **disagree** \| **Neutral** \| \| **Somewhat**  **agree** \| **Agree** \| **Strongly**  **agree** \| \| \| --- \| --- \| --- \| --- \| --- \| --- \| --- \| --- \| --- \| \|  \|  \|  \| 1 \| \| 1 \| 10 \| 5 \| \| \| 0 (0%) \| \| \| 1 (5.9%) \| **16 (94.1%)** \| \| \| \| |  |
| c. Severe edema-like marrow signal – maximum diameter ≥50% of repair tissue diameter | 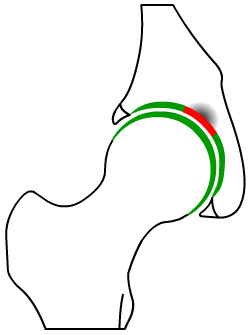 | 10 |
| \| **Strongly**  **disagree** \| **Disagree** \| **Somewhat**  **disagree** \| **Neutral** \| \| **Somewhat**  **agree** \| **Agree** \| **Strongly**  **agree** \| \| \| --- \| --- \| --- \| --- \| --- \| --- \| --- \| --- \| --- \| \|  \|  \|  \|  \| \| 1 \| 11 \| 5 \| \| \| 0 (0%) \| \| \| 0 (0%) \| **17 (100.0%)** \| \| \| \| | \| **Strongly**  **disagree** \| **Disagree** \| **Somewhat**  **disagree** \| **Neutral** \| \| **Somewhat**  **agree** \| **Agree** \| **Strongly**  **agree** \| \| \| --- \| --- \| --- \| --- \| --- \| --- \| --- \| --- \| --- \| \|  \|  \|  \| 1 \| \| 1 \| 9 \| 6 \| \| \| 0 (0%) \| \| \| 1 (5.9%) \| **16 (94.1%)** \| \| \| \| |  |
| d. Subchondral cyst ≥5 mm in longest diameter OR osteonecrosis-like signal | 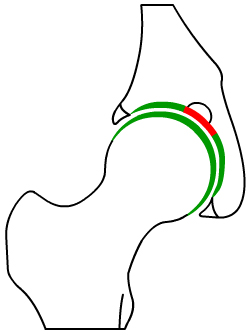 | 0 |
| \| **Strongly**  **disagree** \| **Disagree** \| **Somewhat**  **disagree** \| **Neutral** \| \| **Somewhat**  **agree** \| **Agree** \| **Strongly**  **agree** \| \| \| --- \| --- \| --- \| --- \| --- \| --- \| --- \| --- \| --- \| \|  \|  \|  \|  \| \| 1 \| 9 \| 7 \| \| \| 0 (0%) \| \| \| 0 (0%) \| **17 (100.0%)** \| \| \| \| | \| **Strongly**  **disagree** \| **Disagree** \| **Somewhat**  **disagree** \| **Neutral** \| \| **Somewhat**  **agree** \| **Agree** \| **Strongly**  **agree** \| \| \| --- \| --- \| --- \| --- \| --- \| --- \| --- \| --- \| --- \| \|  \|  \|  \|  \| \| 2 \| 8 \| 7 \| \| \| 0 (0%) \| \| \| 0 (0%) \| **17 (100.0%)** \| \| \| \| |  |

**SECTION 7 - COMMENTS (text coded)**

| d.   - Osteonecrosis be its own category with score of 0 and separate cyst category with a score of 5** - Define “osteonecrosis-like signal” - Don’t use 5mm cut-off for cyst – just present or not |
| --- |
| b. c.   - Note that minor edema and severe is not in terms of intensity of fluid signal but a measure of width of defect. So extent of edema is what you are proposing not grading intensity- so not sure if minor and severe are words to be used. - Cut off for bone marrow edema should be 25% (not 50%) - Criterion c should only be worth 5 points (not 10) |
| Images (b and c):   - The subchondral edema should be the same size in depth but cover only <50% or >50% of the repair |

**SECTION 7 - PROPOSED REVISIONS BASED ON COMMENTS:**

| **Subchondral changes** | **Coronal Plane Image/Diagram** | **Axial Plane Image/Diagram** | **Scoring (Points)** |
| --- | --- | --- | --- |
| 1. No major subchondral changes | 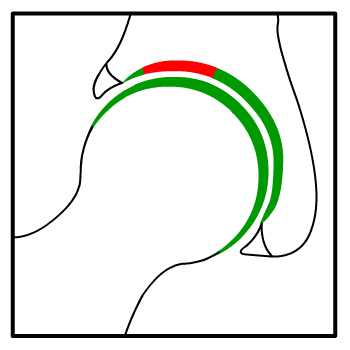 | 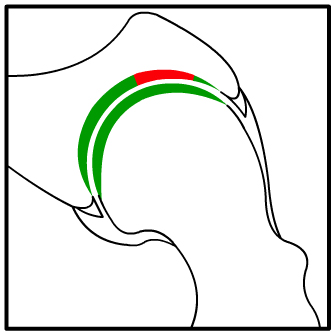 | 20 |
| 1. Minor edema-like marrow signal – maximum diameter <25% of repair tissue diameter | 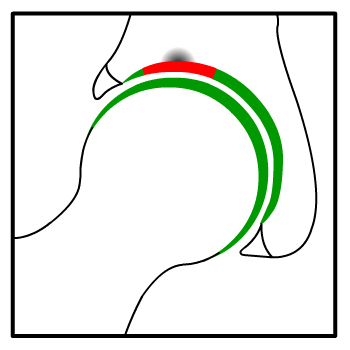 | 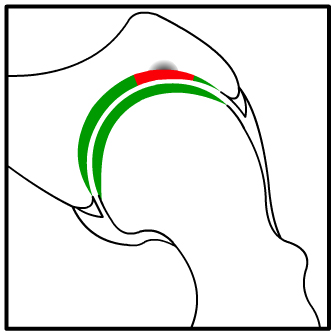 | ~~15~~  0 |
| 1. Severe edema-like marrow signal – maximum diameter ≥25% of repair tissue diameter | 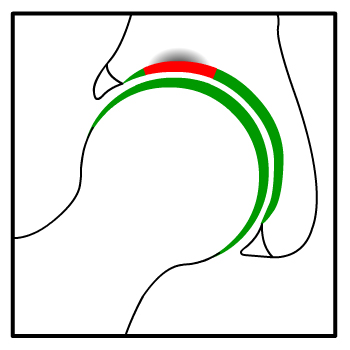 | 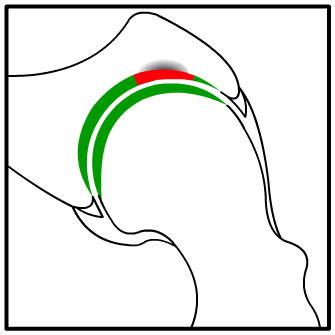 | ~~10~~  -5 |
| 1. Subchondral cyst | 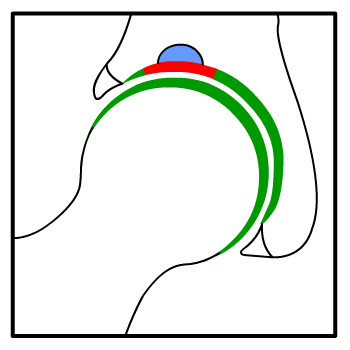 | 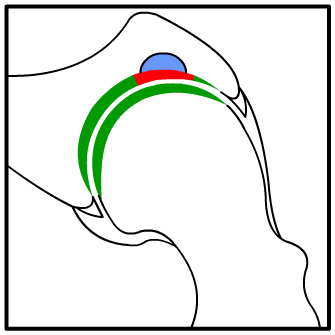 | ~~0~~  -10 |
| 1. Osteonecrosis-like signal | 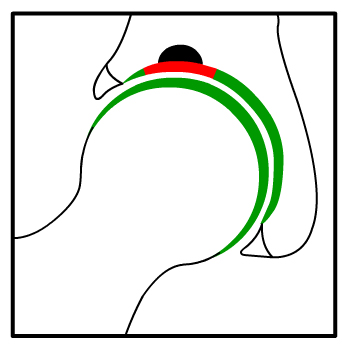 | 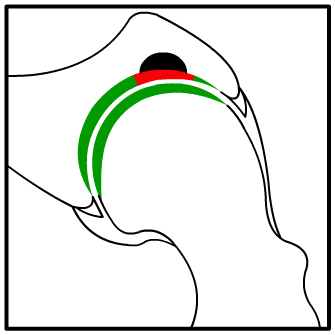 | -20 |

**SECTION 7 - CONSENSUS MEETING QUESTION(S):**

1. For b and c, should 25% or 50% be used as the cut-off value? Or just ‘edema: yes/no’?
2. How should b and c be scored? Zero or a negative score?
3. Is a cyst considered worse than edema? How should these be scored?
4. Remove any mention of osteonecrosis? Too rare to include in score?
5. Vote on changes above.

OTHER GENERAL COMMENTS

| Images:   - More zoomed in, clear - Add MRI examples (not just drawings) |
| --- |
| Views:   - Need biplanar views - Sagittal + coronal (but sagittal could be interpreted using coronal diagrams) – use the worst evaluation for either plane in the score |
